# Supplementary material for: Reconciling Mining with the Conservation of Cave Biodiversity: A Quantitative Baseline to Help Establish Conservation Priorities
Source: PLoS One. 2016 Dec 20;11(12):e0168348. doi: 10.1371/journal.pone.0168348 (PMC5173368; doi:10.1371/journal.pone.0168348)
Supplement: S1 Dataset — (ZIP) [file pone.0168348.s002.zip › Taxa/Serra Norte/SN_2013/N4WS/Lista N4WS-64.pdf]

CAVIDADE N4WS-0064

Classe	Ordem	Fam/Outros	Gên/Outros	Espécie	S-U	U-S	Zon.
Arachnida	Acari	(Oribatida)		sp.1		X	P
Arachnida	Acari	Anystidae	*Erythracarus*	*nasutus*	X	X	P
Arachnida	Acari	Argasidae	*Ornithodoros*	sp.1	X	X	E/P
Arachnida	Acari	Laelapidae		sp.1	X		P
Arachnida	Acari	Otopheidomenidae		sp.1		X	P
Arachnida	Amblypygi	Charinidae	*Charinus*	jovem		2	P
Arachnida	Amblypygi	Phrynidae	*Heterophrynus*	*longicornis*	4	4	E
Arachnida	Araneae	Araneidae		jovem	X		E
Arachnida	Araneae	Ctenidae	*Ctenus*	sp.4	1	1	E
Arachnida	Araneae	Drymusidae	*Drymusa*	*spelunca*	X	X	E/P
Arachnida	Araneae	Mysmenidae		jovem	X		E
Arachnida	Araneae	Ochyroceratidae	*Speocera*	sp.1	X	X	E/P
Arachnida	Araneae	Pholcidae	*Mesabolivar*	*paraensis*	X	X	P
Arachnida	Araneae	Salticidae		sp.1	X	X	E
Arachnida	Araneae	Scytodidae	*Scytodes*	*globula*	3		P
Arachnida	Araneae	Theraphosidae		jovem	3	2	E/P
Arachnida	Araneae	Theridiidae	*Achaearanea*	sp.1	X		E/P
Arachnida	Araneae	Theridiidae	*Theridion*	sp.2	X		E/P
Arachnida	Araneae	Theridiidae		sp.1	X		E
Arachnida	Araneae	Theridiosomatidae	*Plato*	sp.1	X	X	P
Arachnida	Opiliones	Sclerosomatidae	*Prionostema*	sp.1	X	X	P
Arachnida	Opiliones	Stygnidae		jovem		1	E
Arachnida	Palpigradi	Eukoeneniidae	*Eukoenenia*	sp.1	X	X	P
Arachnida	Pseudoscorpiones	Chernetidae	*Spelaeochernes*	sp.1	X	X	P
Arachnida	Pseudoscorpiones	Chthoniidae	*Pseudochthonius*	sp.1	X	X	P
Diplopoda	Polydesmida	Fuhrmannodesmidae		sp.1		X	P
Diplopoda	Polyxenida	Hypogexenidae		sp.1	X		E/P
Diplopoda	Spirostreptida	Pseudonannolenidae	*Pseudonannolene*	sp.1	1	3	P
Entognatha	Collembola	Entomobryidae		sp.4	X		P
Entognatha	Collembola	Isotomidae		sp.1	X		
Entognatha	Collembola	Paronellidae		sp.1	X		
Entognatha	Diplura	Campodeidae		sp.1	X	X	P
Entognatha	Diplura	Projapygidae		sp.1		X	P
Insecta	Blattodea			jovem	2	1	E
Insecta	Coleoptera	Scydmaenidae		sp.12	X		
Insecta	Coleoptera	Tenebrionidae		jovem		X	P
Insecta	Diptera	Ceratopogonidae		sp.	X		
Insecta	Diptera	Chaoboridae		sp.	X		
Insecta	Diptera	Culicidae	*Culex*	sp.	X		
Insecta	Diptera	Phoridae	Metopininae	sp.	X		
Insecta	Diptera	Psychodidae	*Pintomyia*	*gruta*		X	E
Insecta	Diptera	Psychodidae	*Sciopemyia*	*sordellii*	X	X	E/P
Insecta	Diptera	Tipulidae	Tipulinae	sp.	X		E
Insecta	Heteroptera	Reduviidae	Emesinae	sp.2		3	E
Insecta	Heteroptera	Reduviidae	Reduviinae	jovem	1		
Insecta	Heteroptera	Reduviidae	Triatominae	jovem	1		
Insecta	Homoptera	Cixiidae		sp.3	X		
Insecta	Homoptera	Cixiidae		sp.5		X	P
Insecta	Hymenoptera	Formicidae	*Apterostigma*	sp.1	X		

Insecta	Hymenoptera	Formicidae	*Brachymyrmex*	sp.1	X		
Insecta	Hymenoptera	Formicidae	*Dolichoderus*	aff. *bispinosus*	X		E
Insecta	Hymenoptera	Formicidae	*Nylanderia*	sp.1	X		
Insecta	Hymenoptera	Formicidae	*Nylanderia*	sp.3		X	E/P
Insecta	Hymenoptera	Formicidae	*Pachycondyla*	*striata*		1	P
Insecta	Hymenoptera	Formicidae	*Solenopsis*	sp.1	X		
Insecta	Hymenoptera	Formicidae	*Wasmannia*	sp.1	X		
Insecta	Isoptera	Termitidae	*Diversitermes*	sp.		X	E
Insecta	Isoptera	Termitidae	*Nasutitermes*	sp.	X	X	E/P
Insecta	Lepidoptera	Noctuoidea		sp.1	1		E
Insecta	Lepidoptera	Noctuoidea		sp.2		X	E
Insecta	Lepidoptera	Noctuoidea		sp.4		X	P
Insecta	Orthoptera	Phalangopsidae	*Paraclodes*	sp.	8		E
Insecta	Orthoptera	Phalangopsidae	*Phalangopsis*	sp.	28	22	E/P
Insecta	Psocoptera	Epipsocidae	*Epipsocus*	sp.2		X	P
Insecta	Psocoptera	Manicapsocidae	*Nothoentomum*	sp.1		X	E
Insecta	Psocoptera	Psyllipsocidae	*Psyllipsocus*	sp.2		X	P
Insecta	Thysanura	Nicoletiidae		sp.1	X		
Gastropoda	Pulmonata	Bulimulidae	*Naesiotus*	sp.		X	P
Gastropoda	Pulmonata	Subulinidae	*Lamellaxis*	sp.		X	P
Gastropoda	Pulmonata	Systrophiidae	*Happia*	sp.		X	E/P
Malacostraca	Isopoda	Philosciidae		sp.1		X	P
Malacostraca	Isopoda	Philosciidae		sp.2	X	X	P
Symphyla		Scutigerellidae	*Hanseniella*	sp.1	X		P
Amphibia	Anura	Strabomantidae	*Pristimantis*	cf. *fenestratus*	3		E/P
Amphibia	Anura			sp.3	1		E
Mammalia	Chiroptera	Emballonuridae	*Peropteryx*	sp.		2	
Mammalia	Chiroptera	Phyllostomidae	*Carollia*	*perspicillata*	4	4	P
Mammalia	Chiroptera	Phyllostomidae	Glossophaginae	sp.		4	
